# Retinoic acid has different effects on UCP1 expression in mouse and human adipocytes

**DOI:** 10.1186/1471-2121-14-41

**Published:** 2013-09-23

**Authors:** Maria Murholm, Marie S Isidor, Astrid L Basse, Sally Winther, Cathrine Sørensen, Jonas Skovgaard-Petersen, Maja M Nielsen, Aina S Hansen, Bjørn Quistorff, Jacob B Hansen

**Affiliations:** 1 Department of Biology, University of Copenhagen, Universitetsparken 13, DK-2100 Copenhagen Ø, Denmark; 2 Department of Biomedical Sciences, University of Copenhagen, Blegdamsvej 3, DK-2200 Copenhagen N, Denmark

**Keywords:** Adipogenesis, ATRA, Brown adipocyte, UCP1, White adipocyte

## Abstract

**Background:**

Increased adipose thermogenesis is being considered as a strategy aimed at preventing or reversing obesity. Thus, regulation of the uncoupling protein 1 (UCP1) gene in human adipocytes is of significant interest. Retinoic acid (RA), the carboxylic acid form of vitamin A, displays agonist activity toward several nuclear hormone receptors, including RA receptors (RARs) and peroxisome proliferator-activated receptor δ (PPARδ). Moreover, RA is a potent positive regulator of UCP1 expression in mouse adipocytes.

**Results:**

The effects of all-*trans* RA (ATRA) on UCP1 gene expression in models of mouse and human adipocyte differentiation were investigated. ATRA induced UCP1 expression in all mouse white and brown adipocytes, but inhibited or had no effect on UCP1 expression in human adipocyte cell lines and primary human white adipocytes. Experiments with various RAR agonists and a RAR antagonist in mouse cells demonstrated that the stimulatory effect of ATRA on UCP1 gene expression was indeed mediated by RARs. Consistently, a PPARδ agonist was without effect. Moreover, the ATRA-mediated induction of UCP1 expression in mouse adipocytes was independent of PPARγ coactivator-1α.

**Conclusions:**

UCP1 expression is differently affected by ATRA in mouse and human adipocytes. ATRA induces UCP1 expression in mouse adipocytes through activation of RARs, whereas expression of UCP1 in human adipocytes is not increased by exposure to ATRA.

## Background

Mammals have two types of fat, white and brown adipose tissue (WAT and BAT, respectively), that carry out essentially opposite functions in whole body energy metabolism [1,2]. White adipocytes are specialized in energy storage and their content of triglyceride constitutes the largest energy reserve of the body. Contrary, brown adipocytes have a high capacity for energy dissipation through adaptive thermogenesis due to the presence of the brown adipocyte-specific uncoupling protein 1 (UCP1) in the inner membrane of the abundant mitochondria. BAT has been shown to counteract obesity and is important for rodents to defend their body temperature in response to prolonged cold exposure [1]. Brown-like adipocytes expressing UCP1 appear in some rodent WAT depots after cold exposure or treatment with β-adrenergic agonists [3,4]. Recent studies have suggested a negative correlation between body mass index and the amount of active BAT in humans [5]. Strategies aiming at increasing levels of UCP1 in WAT have become of interest as reduced expression of brown adipocyte-enriched genes in WAT is associated with obesity and type 2 diabetes in humans [6-8].

Retinoic acid (RA) is a derivative of vitamin A that affects cellular growth, differentiation and apoptosis in various embryonic and adult tissues [9,10]. All-*trans* RA (ATRA) has been reported being an agonist for multiple nuclear receptors, including RA receptors (RARs) [11,12], peroxisome proliferator-activated receptor δ (PPARδ, also designated PPARβ) [13], testicular orphan receptor 4 (TR4) [14] and chicken ovalbumin upstream promoter transcription factor II (COUP-TFII) [15]. It has been proposed that PPARδ mediates part of the metabolic effects of ATRA [16]. Additionally, ATRA has been shown to regulate gene expression in a nongenomic manner [17]. However, it is believed that most effects of ATRA are mediated by RARs that upon heterodimerization with retinoid X receptors control gene expression through binding to RA response elements in regulatory regions of target genes [9,18].

High concentrations of ATRA inhibit differentiation of 3T3-L1 white preadipocytes and C3H10T½ mesenchymal stem cells [19-21], whereas low concentrations have been shown to stimulate white adipogenesis of Ob1771 cells [22]. The inhibition of adipogenesis by ATRA is mediated by RARs and is linked to suppression of CCAAT/enhancer-binding protein β activity and induction of anti-adipogenic genes [19,21,23].

The UCP1 gene of mice, rats and humans contains RAR-responsive elements in its enhancer region and ATRA has been shown to promote UCP1 expression and oxidative metabolism in cultured rodent adipocytes [24-31]. Moreover, treatment of mice with ATRA causes increased expression of UCP1 in WAT and BAT [17,28,31,32].

In the present study we compared the response of mouse and human preadipocytes and mature adipocytes to ATRA, with emphasis on the effects on differentiation and UCP1 expression. In addition, we have studied the importance of RARs, PPARδ and PGC-1α for the regulation of UCP1 expression by ATRA. We find that ATRA increases UCP1 expression in all mouse adipocyte models studied, including 3T3-L1 white adipocytes, and that this induction is mediated by RARs and is independent of PPARδ and PGC-1α. Finally, ATRA does not increase UCP1 expression in any of the human adipocytes examined in this study.

## Results

### Exposure of differentiating mouse adipocytes to ATRA increases UCP1 expression

In order to examine the effects of ATRA on differentiating mouse adipocytes, we exposed four cell models of adipogenesis to a range of ATRA concentrations (10 nM to 10 μM) throughout the course of the differentiation process, i.e. between days −2 (the time of confluence) and 8 (designated chronic exposure). Gene expression was analyzed at day 8. We estimated the degree of differentiation by measuring mRNA levels of the adipocyte marker gene fatty acid-binding protein 4 (FABP4, also designated aP2). Expression of the brown fat-specific UCP1 gene was determined at both the mRNA and protein levels, and the expression of RARβ was used to estimate the degree of activation of RARs, as the RARβ gene is responsive to retinoids [33,34]. The cells used were 3T3-L1 preadipocytes, wild-type (WT) mouse embryo fibroblasts (MEFs) and the mesenchymal stem cell line C3H10T½ as models of white adipocyte differentiation [35,36], and MEFs lacking a functional retinoblastoma gene (Rb−/−) as a model of brown adipocyte differentiation [35].

**Figure 1:**
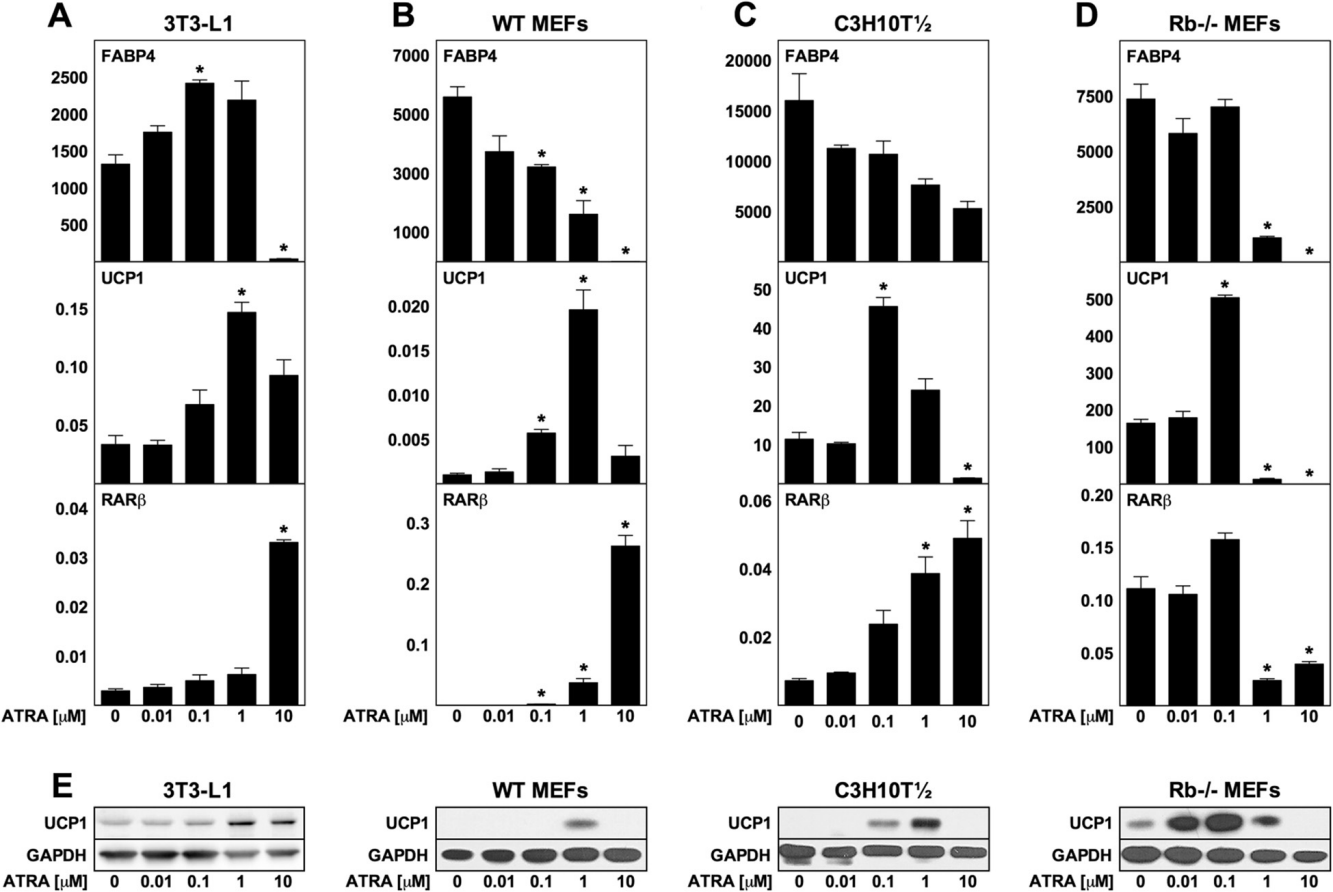
**Chronic treatment with ATRA induces UCP1 expression in differentiating mouse adipocytes.** 3T3-L1 cells, WT MEFs, C3H10T½ cells and Rb−/− MEFs were treated with ATRA from day −2 to day 8 in the concentrations indicated. Total RNA and protein were harvested at day 8 and analyzed by RT-qPCR and immunoblotting, respectively. Relative mRNA expression levels of FABP4, UCP1 and RARβ were determined by normalization to expression levels of TATA-binding protein (TBP). **(A)** 3T3-L1 cells. **(B)** WT MEFs. **(C)** C3H10T½. **(D)** Rb−/− MEFs. **(E)** Protein levels of UCP1 with GAPDH used as a loading control. **(A**-**D)** Data represents mean + SEM (n = 3). *, p < 0.05 versus vehicle-treated cells.

RARβ expression increased dose-dependently in response to treatment with ATRA in 3T3-L1, WT MEFs and C3H10T½ cells, whereas the same pattern was not observed in Rb−/− MEFs (Figure 1A-D). A dose-dependent reduction of FABP4 expression was seen in WT MEFs, C3H10T½ and Rb−/− MEFs, whereas only the highest level of ATRA (10 μM) blocked adipose conversion of 3T3-L1 cells (Figure 1A-D). ATRA in intermediate concentrations (1 μM in 3T3-L1, 0.1 μM and 1 μM in WT MEF-derived adipocytes and 0.1 μM in C3H10T½ and Rb−/− adipocytes) induced expression of UCP1 at both mRNA and protein levels, with a maximum fold induction of mRNA levels of 4, 18, 4 and 3 in 3T3-L1, WT MEF-derived, C3H10T½ and Rb−/− MEF-derived adipocytes, respectively (Figure 1A-E). Although ATRA in intermediate concentrations induced expression of UCP1 in all cases, the absolute level of UCP1 varied substantially between the cell models, being highest in Rb−/− adipocytes and lowest in WT MEF-derived adipocytes. In summary, chronic exposure to high concentrations of ATRA inhibits adipocyte differentiation, whereas intermediate concentrations cause increased expression of UCP1, even in 3T3-L1 white adipocytes.

### Exposing mature mouse adipocytes to ATRA enhances expression of UCP1

**Figure 2:**
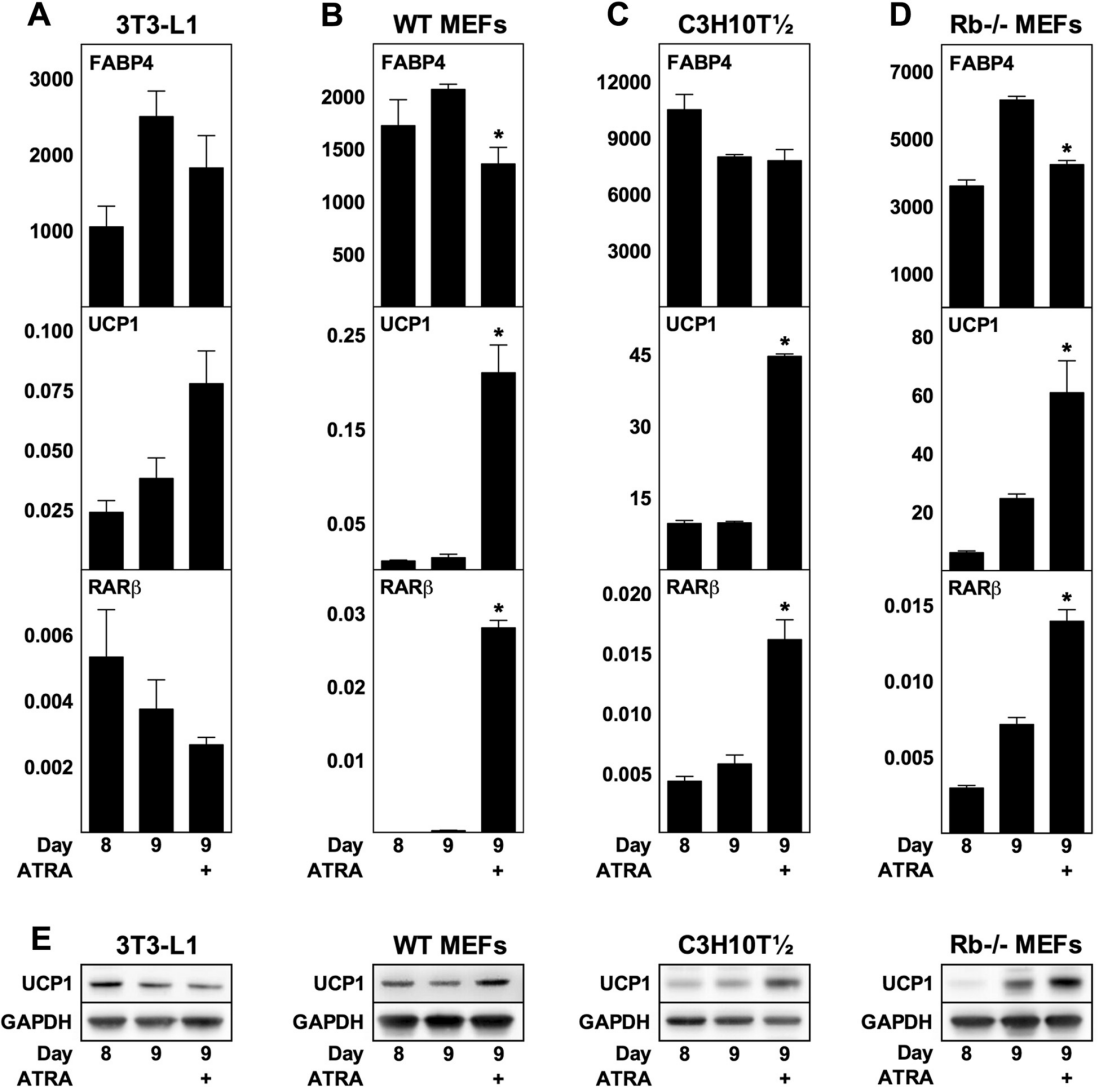
**Acute exposure of mature mouse adipocytes to ATRA induces UCP1 expression.** ATRA (1 μM) was supplemented at day 8. Total RNA and protein were harvested after 24 h and analyzed by RT-qPCR and immunoblotting, respectively. Relative mRNA expression levels of FABP4, UCP1 and RARβ were determined by normalization to levels of TBP. **(A)** 3T3-L1. **(B)** WT MEFs. **(C)** C3H10T½. **(D)** Rb−/− MEFs. **(E)** Protein levels of UCP1 with GAPDH used as a loading control. **(A**-**D)** Data represents mean + SEM (n = 3). *, p < 0.05 versus vehicle-treated cells at day 9.

In addition to the chronic exposure to ATRA described above, we also analyzed the effects of exposing mature mouse adipocytes to ATRA at day 8 (designated acute exposure). In this case, we used a fixed concentration of ATRA (1 μM) and harvested RNA and protein after 24 h (i.e. at day 9). Expression of UCP1 and RARβ increased in response to ATRA compared to vehicle in WT and Rb−/− MEF-derived and C3H10T½ adipocytes, but not in 3T3-L1 adipocytes, with UCP1 mRNA levels increasing 15-, 4.6- and 2.5-fold, respectively, in the former three cell models (Figure 2A-D). Levels of UCP1 protein mirrored the levels of UCP1 mRNA (Figure 2E). FABP4 mRNA levels were slightly reduced by the acute exposure to ATRA in MEF-derived adipocytes, but not in 3T3-L1 and C3H10T½ adipocytes.

### RARs mediate the effects of ATRA

**Figure 3:**
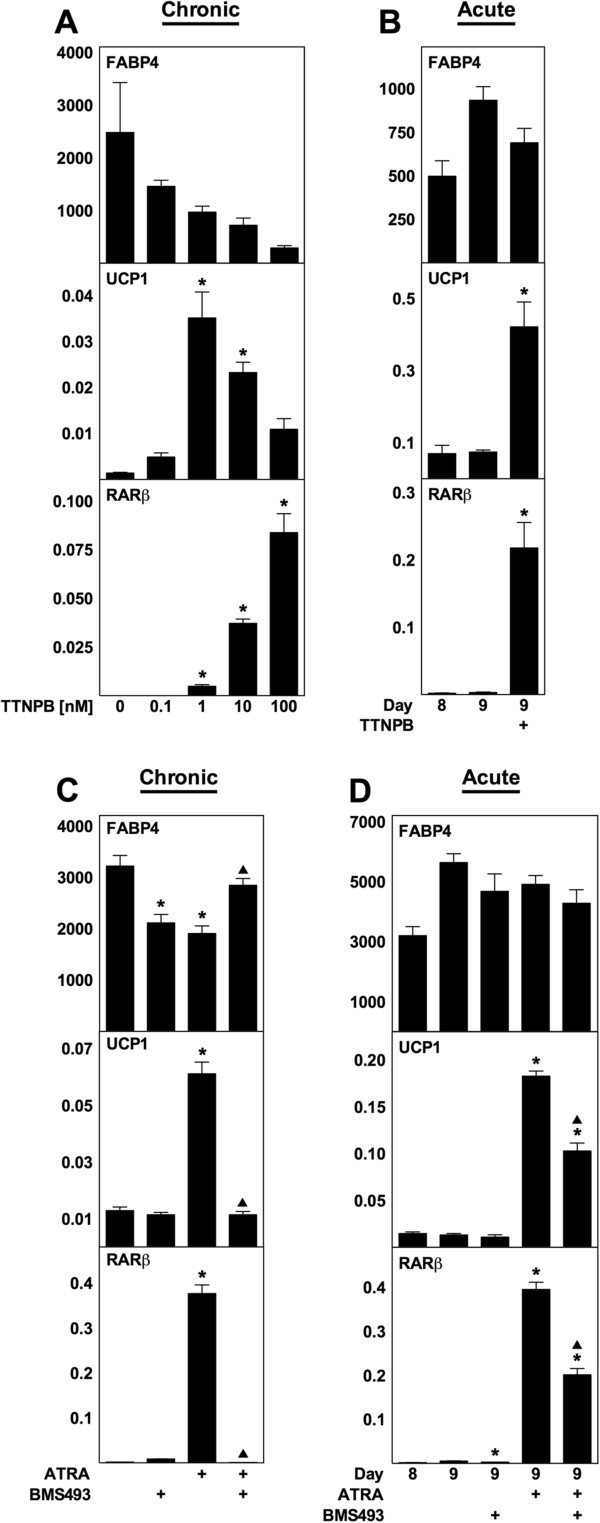
**TTNPB mimics and BMS493 inhibits the effects of ATRA in WT MEFs.** Total RNA was harvested at the indicated days (day 8 in panels A and C) and analyzed by RT-qPCR. Relative mRNA expression levels of FABP4, UCP1 and RARβ were determined by normalization to TBP. **(A)** TTNPB was supplemented to differentiating cells from day −2 to day 8 at the concentrations indicated. **(B)** TTNPB (1 nM) was acutely supplemented to mature adipocytes (day 8) and harvested after 24 h. **(C)** Treatment with BMS493 (1 μM) and/or ATRA (1 μM) from day −2 to day 8. **(D)** Acute supplementation of BMS493 (1 μM) and/or ATRA (1 μM) to mature adipocytes from day 8 and harvested after 24 h. Data represents mean + SEM (n = 3). *, p < 0.05 versus vehicle-treated cells. ^▲^, p < 0.05 versus ATRA-treated cells.

As ATRA has been shown to bind and activate a range of nuclear receptors, we wished to identify the relevant targets of ATRA mediating the effects observed above. Therefore, we treated WT MEFs with TTNPB, a pan-RAR agonist not displaying agonist activity toward PPARδ [13], using the two same experimental setups described above. Both chronic application of TTNPB (Figure 3A) and acute application of TTNPB to mature adipocytes (Figure 3B) resulted in expression patterns of FABP4, UCP1 and RARβ similar to those observed upon treatment with ATRA (see Figures 1 and 2). Expression levels of UCP1 reached a peak induction of 25-fold in chronically treated adipocytes (1 nM TTNPB) (Figure 3A) and 6-fold after treatment of mature adipocytes with 1 nM TTNPB for 24 h (Figure 3B). Next, we investigated if the pan-RAR antagonist BMS493 could inhibit the UCP1-inducing effects of ATRA. When applied chronically during differentiation (Figure 3C) and acutely to mature adipocytes (Figure 3D), BMS493 eliminated and blunted, respectively, the enhanced expression of UCP1 caused by ATRA. These results strongly suggest that the action of ATRA is mediated through RARs.

**Figure 4:**
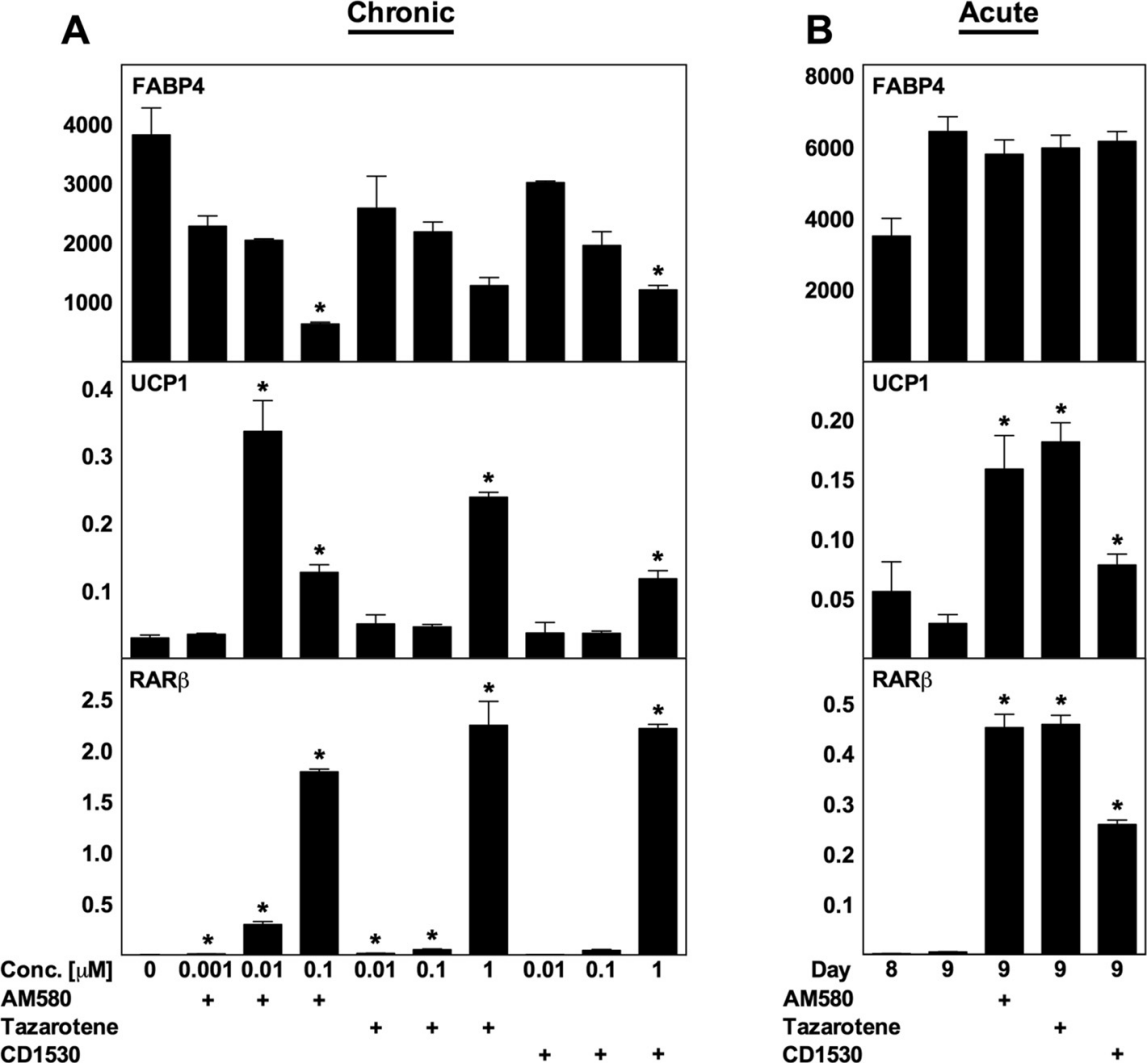
**RAR subtype-selective ligands induce UCP1 expression in WT MEFs.** Total RNA was harvested at day 8 **(panel A)** or at the indicated days **(panel B)** and analyzed by RT-qPCR. Relative mRNA expression levels of FABP4, UCP1 and RARβ were determined by normalization to TBP. **(A)** RAR subtype-selective ligands AM580, tazarotene and CD1530 were supplemented from day −2 to day 8 at the concentrations indicated. **(B)** RAR subtype-selective ligands AM580 (1 μM), tazarotene (1 μM) and CD1530 (1 μM) were acutely supplemented to mature adipocytes from day 8 and harvested after 24 h. Data represents mean + SEM (n = 3). *, p < 0.05 versus vehicle-treated cells.

To delineate which of the three RAR isoforms that are responsible for the upregulation of UCP1, we used three RAR subtype-selective agonists: AM580 (RARα selective); tazarotene (RARβ/γ selective) and CD1530 (RARγ selective). All three agonists caused an induction of UCP1 expression when administered chronically to differentiating WT MEFs (Figure 4A). AM580 and tazarotene induced the highest fold increase in UCP1 expression (11- and 8-fold, respectively). A dose-dependent increase in RARβ mRNA and a reduction of FABP4 mRNA levels were observed for all three agonists (Figure 4A). Notably, as AM580 has a 30-40-fold selectivity for RARα compared to RARβ and RARγ [37], the observation that 10 nM AM580 was sufficient to increase UCP1 levels suggests that activation of RARα is sufficient (Figure 4A). However, tazarotene and CD1530 were also able to increase UCP1 levels despite a low affinity for RARα, indicating that activation of any RAR subtype is capable of increasing UCP1 expression in mouse adipocytes.

When the three agonists were acutely supplemented to mature WT MEF-derived adipocytes, UCP1 mRNA levels were significantly induced after 24 h, with AM580 and tazarotene displaying the most potent effect (Figure 4B).

### PPARδ activation does not increase UCP1 expression in MEF-derived white adipocytes

**Figure 5:**
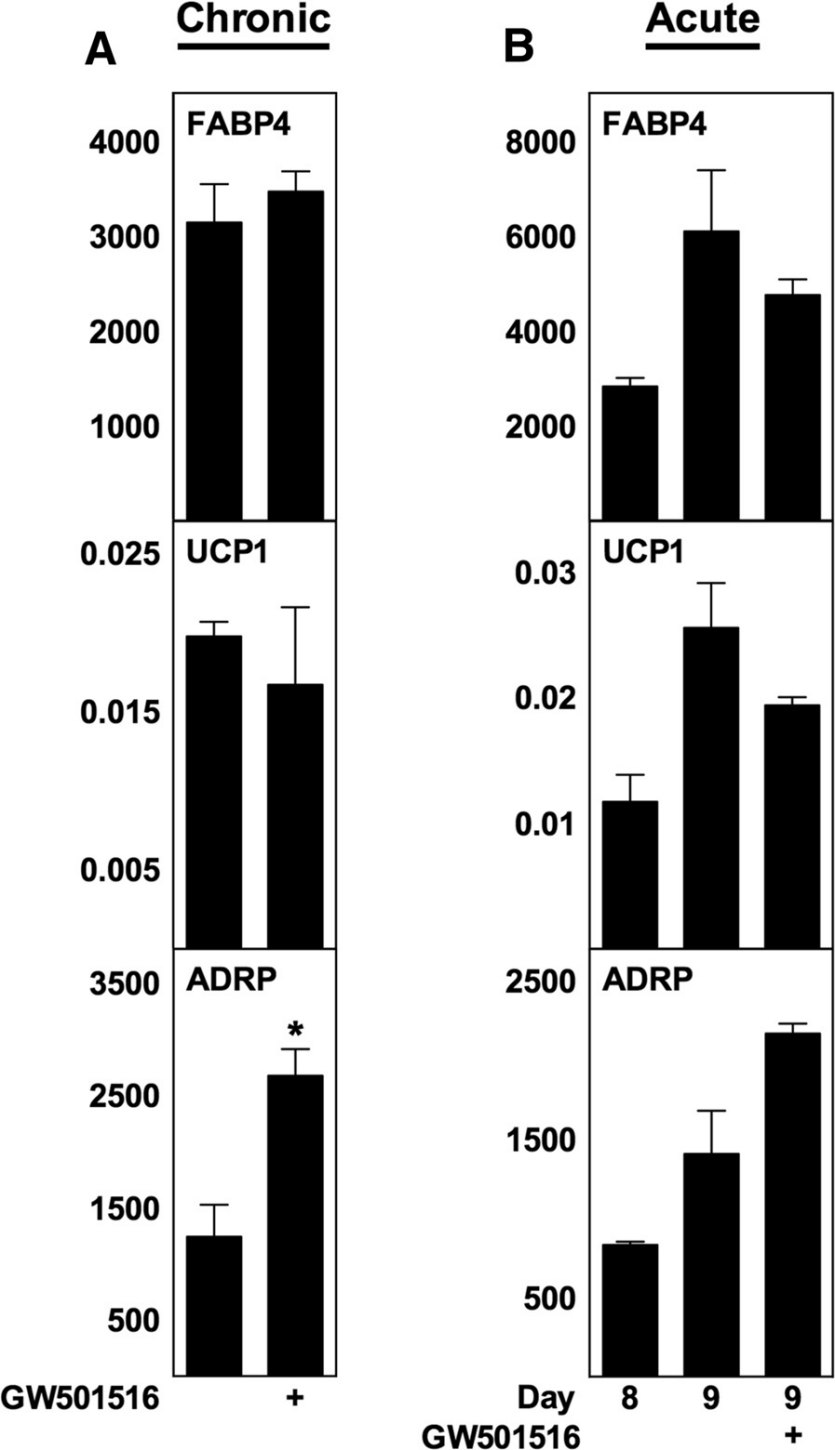
**Activation of PPARδ in WT MEFs does not increase UCP1 expression.** Total RNA was harvested at day 8 **(panel A)** or at the indicated days **(panel B)** and analyzed by RT-qPCR. Relative mRNA expression levels of FABP4, UCP1 and ADRP were determined by normalization to TBP. **(A)** Treatment with the PPARδ agonist GW501516 (1 μM) from day −2 to day 8. **(B)** GW501516 (1 μM) was acutely supplemented to mature adipocytes from day 8 and harvested after 24 h. Data represents mean + SEM (n = 3). *, p < 0.05 versus vehicle-treated cells.

As mentioned, ATRA can bind to PPARδ and increase its transcriptional activity [13,38], and activation of PPARδ is believed to regulate thermogenic gene expression in adipose tissue [39,40]. To determine if activation of PPARδ mimics the effects observed with ATRA, we exposed WT MEFs to the PPARδ agonist GW501516 either during differentiation (Figure 5A) or acutely to mature adipocytes (Figure 5B). GW501516 did not change either UCP1 or FABP4 mRNA levels in either case (Figure 5A and B). The PPARδ target gene adipose differentiation-related protein (ADRP, also designated adipophilin and perilipin 2) [41] was significantly upregulated when GW501516 was added chronically (Figure 5A).

### The effects of ATRA on UCP1 expression is not dependent on PGC-1α

**Figure 6:**
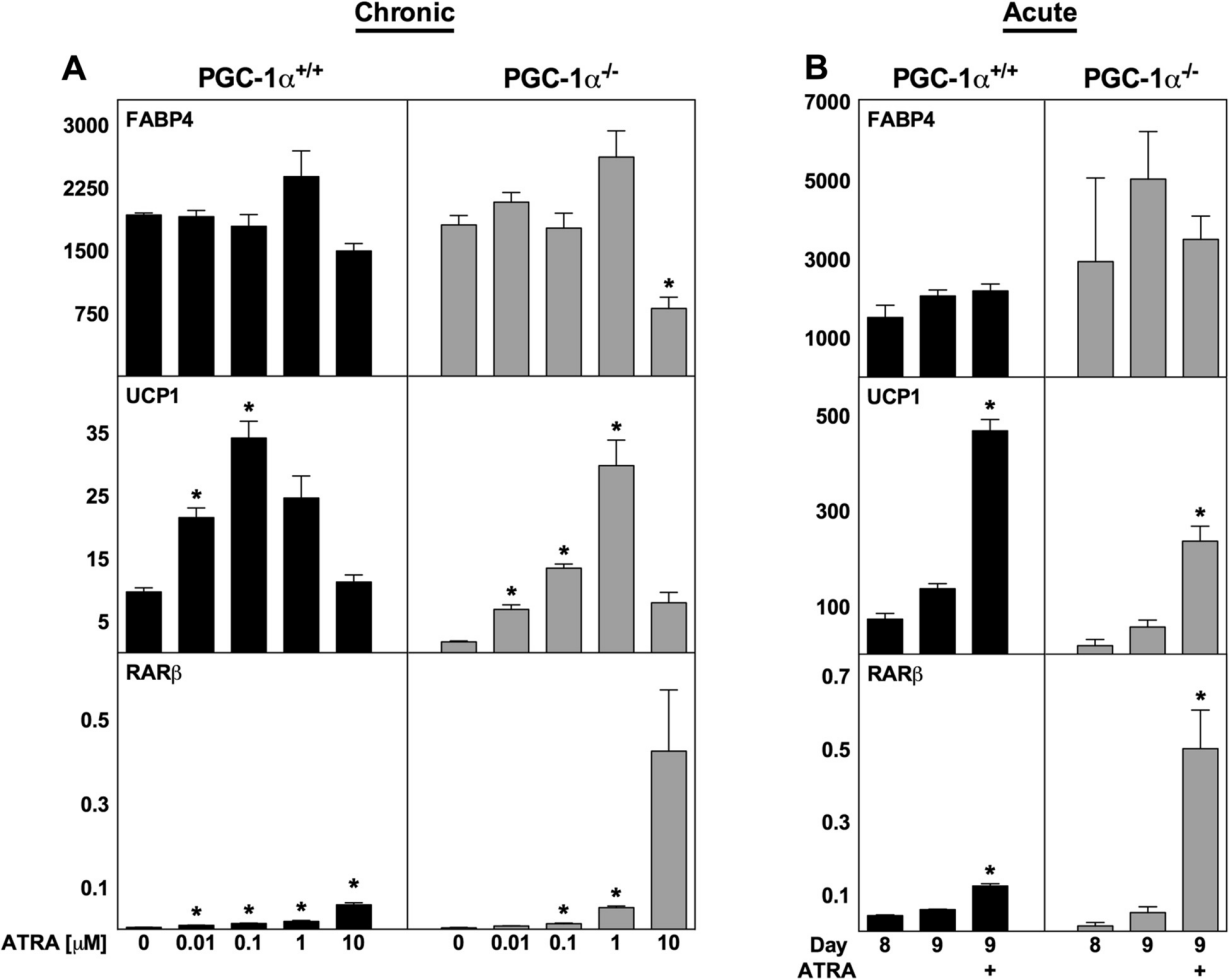
**ATRA enhances UCP1 expression independent of PGC-1α.** Total RNA was harvested at day 8 **(panel A)** or at the indicated days **(panel B)** and analyzed by RT-qPCR. Relative mRNA expression levels of FABP4, UCP1 and RARβ were determined by normalization to TBP. **(A)** ATRA was supplemented to differentiating PGC-1α+/+ and PGC-1α−/− brown preadipocytes from day −2 to day 8 at the concentrations indicated. **(B)** ATRA (1 μM) was acutely supplemented to mature PGC-1α+/+ and PGC-1α−/− adipocytes from day 8 and harvested after 24 h. Data represents mean + SEM (n = 3). *, p < 0.05 versus vehicle-treated cells.

PGC-1α is a metabolically regulated transcriptional coactivator that is known to induce UCP1 levels in adipocytes and to interact with RARα in an ATRA-dependent manner [42]. To investigate whether PGC-1α is required for the ATRA-mediated effects, we compared immortalized brown preadipocyte cell lines from WT and PGC-1α-deficient mice [43]. ATRA was supplemented either chronically during differentiation or acutely to mature adipocytes. UCP1 expression was increased at intermediate concentrations of ATRA in the chronically treated PGC-1α+/+ and PGC-1α−/− adipocytes (Figure 6A). The highest level of UCP1 was observed with 0.1 μM ATRA in WT adipocytes (3.5-fold above vehicle), but with 1 μM ATRA in PGC-1α−/− adipocytes (17-fold above vehicle). Of notice, basal expression of UCP1 was lower in PGC-1α-deficient fat cells (Figure 6A and B). A minor decrease in FABP4 expression with 10 μM ATRA and a dose-dependent increase in RARβ expression were observed (Figure 6A).

Exposing mature PGC-1α+/+ and PGC-1α−/− adipocytes to 1 μM ATRA elicited significant induction of UCP1 after 24 h (Figure 6B). Thus, enhanced expression of UCP1 caused by ATRA does not require PGC-1α.

### ATRA inhibits human adipocyte differentiation in a dose-dependent manner and does not increase UCP1 expression

**Figure 7:**
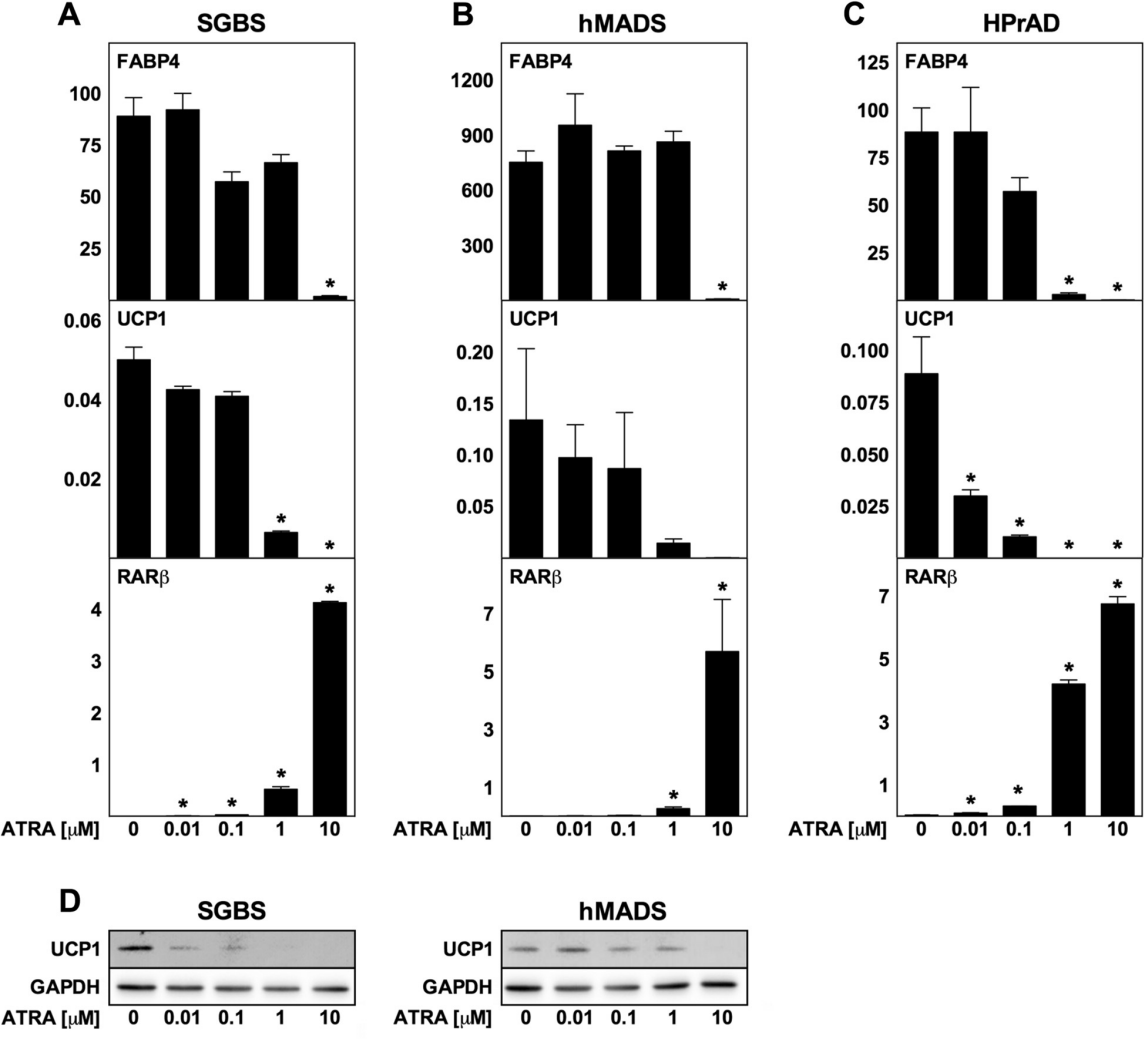
**Chronic treatment with ATRA does not increase UCP1 expression in differentiating SGBS, hMADS and primary human adipocytes.** Total RNA was harvested at the indicated days (day 12 in panel A and B) and analyzed by RT-qPCR. Relative mRNA expression levels of FABP4, UCP1 and RARβ were determined by normalization to TBP. ATRA was supplemented to differentiating SGBS cells **(A)**, hMADS cells **(B)** and primary human white preadipocytes (HPrAD) **(C)** from day −2 to day 12 at the concentrations indicated. **(D)** Protein levels of UCP1 with GAPDH used as a loading control in SGBS and hMADS adipocytes. **(A**-**C)** Data represents mean + SEM (n = 3). *, p < 0.05 versus vehicle-treated cells.

**Figure 8:**
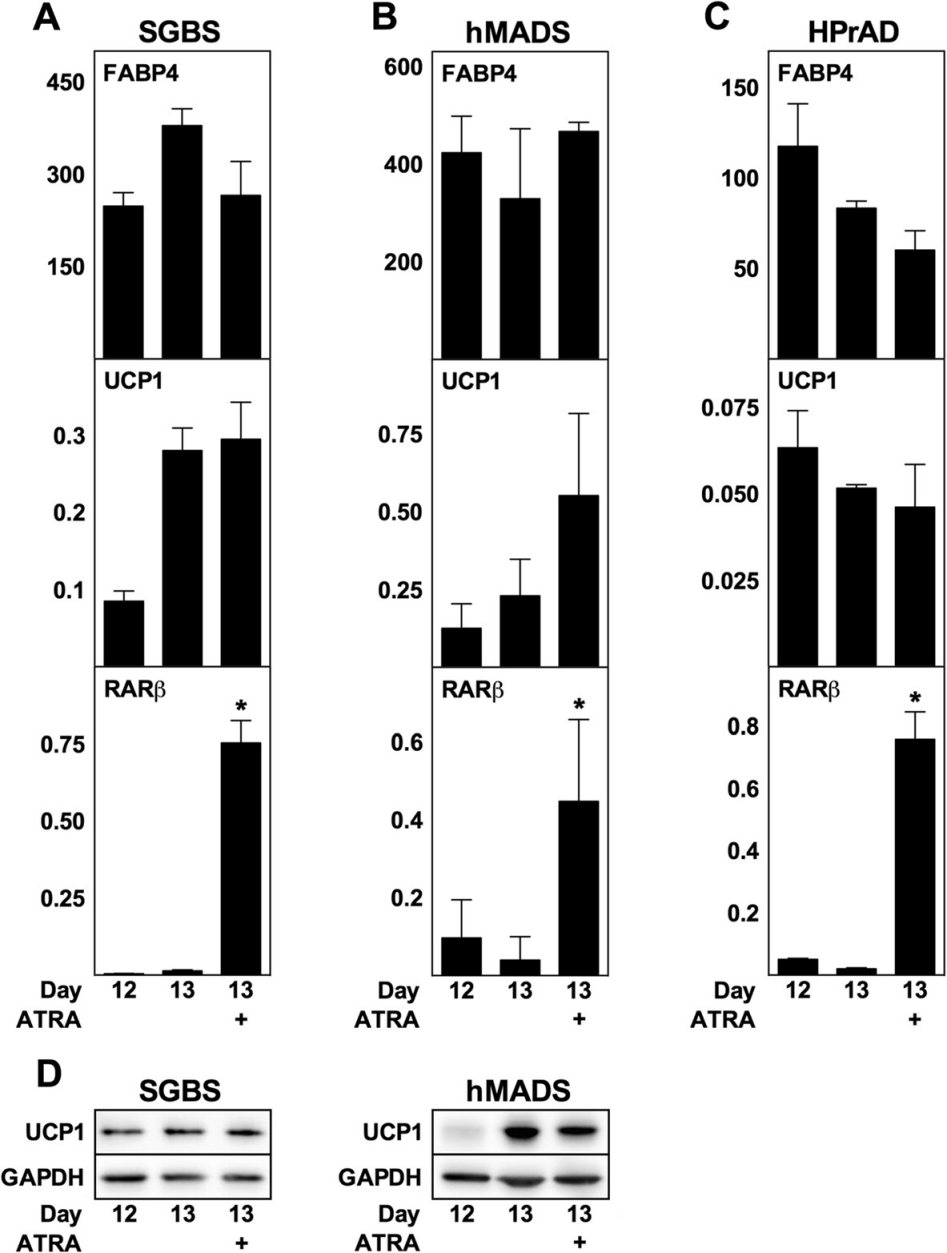
**Acute exposure of mature human adipocytes to ATRA does not induce UCP1 expression.** ATRA (1 μM) was supplemented to mature SGBS adipocytes **(A)**, hMADS adipocytes **(B)** and primary human white preadipocytes (HPrAD) **(C)** from day 12 and harvested after 24 h. **(D)** Protein levels of UCP1 with GAPDH used as a loading control in SGBS and hMADS adipocytes. **(A**-**C)** Data represents mean + SEM (n = 3). *, p < 0.05 versus vehicle-treated cells.

To investigate if chronic treatment with ATRA had the same effects on human adipocytes as observed with mouse adipocytes, we exposed the human white preadipocyte cell line SGBS [44], human multipotent adipose-derived stem cells (hMADS) [45,46] and primary human white preadipocytes to various concentrations of ATRA during differentiation (Figure 7A-C). The human adipocytes were considered mature on day 12, and not on day 8 as the mouse adipocytes, as their morphological differentiation into adipocytes was substantially slower. High concentrations of ATRA inhibited differentiation of SGBS (10 μM), hMADS (10 μM) and primary preadipocytes (1 and 10 μM), as estimated by expression of FABP4 mRNA. RARβ expression increased dose-dependently in all three cell models (Figure 7A-C). Interestingly, the low basal UCP1 mRNA levels in SGBS and hMADS adipocytes did not increase at any concentration of ATRA compared with vehicle-treated cells and were lower with 1 and 10 μM ATRA (Figure 7A and B). Notably, FABP4 expression was not affected by 1 μM ATRA. Basal expression of UCP1 in primary preadipocytes exposed to ATRA during differentiation was strongly inhibited by ATRA, an inhibition observed even at the lowest concentration used (10 nM) (Figure 7C). Levels of UCP1 protein were determined in SGBS and hMADS adipocytes and confirmed that ATRA did not increase UCP1 levels (Figure 7D). Acute exposure of mature adipocytes to 1 μM ATRA did not result in any significant changes in mRNA levels of FABP4 or mRNA and protein levels of UCP1 compared to vehicle treatment in SGBS, hMADS or primary cells (Figure 8). Basal UCP1 mRNA expression was undetectable in primary human white adipocytes from another donor and expression remained undetectable after treatment with ATRA (data not shown). In summary, ATRA does not increase expression of UCP1 in the human adipocyte models studied here.

## Discussion

In this study, we report the effects of ATRA on differentiation and UCP1 expression in various mouse and human adipocytes. We find that high concentrations of ATRA inhibit mouse and human adipogenesis, whereas lower concentrations enhance UCP1 expression in mouse, but not in human, adipocytes. In addition, we show that the effects of ATRA are mediated by RARs and not by PPARδ or other ATRA-activated nuclear receptors. Moreover, the enhanced expression of UCP1 in response to ATRA is independent of PGC-1α.

Adipocyte and adipose tissue function are impacted by ATRA [47,48]. Expression of UCP1 is reduced in BAT of mice fed vitamin A-depleted feed [17,49] and exogenous ATRA enhances expression of UCP1 in both WAT and BAT of mice and rats [16,17,28,32,49,50]. Expression of UCP1 is induced by ATRA in primary brown adipocytes from mice and rats as well as in mouse brown adipocyte cell lines [24,26,28,29,51-53]. Moreover, UCP1 expression is strongly induced in MEF-derived white adipocytes [31]. Contrary, ATRA has been reported not to induce expression of UCP1 in mature 3T3-L1 adipocytes and mouse primary white adipocytes [16,30,54]. Exposure to ATRA leads to activation of p38 mitogen-activated protein kinase (MAPK), an activation that is required for full induction of UCP1 expression by ATRA [29,31].

ATRA has been reported to activate three nuclear receptors besides RARs, namely PPARδ, COUP-TFII and TR4. The induction of UCP1 observed in the mouse cells applied in this study is unlikely to be mediated by COUP-TFII and TR4, as the app. EC_50_ of ATRA are 20 μM [15] and 24 μM [14], respectively, which is 20 to 240 times higher than the concentrations inducing UCP1. Although the EC_50_ of ATRA for PPARδ is much lower than for COUP-TFII and TR4 (app. 200 nM) [13], PPARδ is not mediating the effects of ATRA either. Firstly, the RAR agonist TTNPB mimics the effects of ATRA (see Figure 3), but does not bind to PPARδ [13]. Secondly, a potent PPARδ agonist does not enhance expression of UCP1 (see Figure 5). Thirdly, a RAR antagonist attenuates the effects of ATRA (see Figure 3). In this study, we have not addressed the potential involvement of nongenomic effects of ATRA, e.g. activation of p38 MAPK and the cell surface receptor responsible for retinol uptake called stimulated by retinoic acid gene 6 [55].

At intermediate concentrations of ATRA, we consistently observe an induction of UCP1 expression in mouse adipocytes. This does not only occur in the mouse cells shown here, but was also observed with WT-1 brown adipocytes [56,57] and 3T3-F442A white adipocytes (data not shown). Thus, our results demonstrate that ATRA can cause an induction of UCP1 expression in white adipocyte cell models of mouse origin. It is tempting to speculate that exposure to ATRA will cause white preadipocytes and mature adipocytes to transdifferentiate into brown-like adipocytes *in vitro*. However, in order to confirm if a transdifferentiation event has taken place in our study, a more detailed gene expression analysis is required combined with a characterization of mitochondrial function.

Using three cell models of human origin, SGBS and hMADS cells as well as primary subcutaneous adipocytes from two different donors, we failed to detect an induction of UCP1 expression by ATRA (see Figures 7 and 8). hMADS cells have been proposed to represent brown or brown-like adipocytes, the latter due to the induction of UCP1 expression in response to prolonged culture in the presence of rosiglitazone [58,59] or upon treatment with atrial natriuretic peptide [60]. Despite being considered white fat cells, SGBS and primary subcutaneous human adipocytes have the ability to induce expression of UCP1 in response to genetic manipulation [61,62]. Thus, the lack of effect of ATRA in the human adipocyte models studied here cannot be explained by an inherent inability to induce expression of UCP1. Consistently, to our knowledge, an induction of the endogenous human UCP1 gene by ATRA has never been reported. Nevertheless, we cannot rule out that the lack of response in our study is due to the experimental setup or the human cell models used. In particular, it remains to be shown if primary human brown adipocytes respond to ATRA by increasing UCP1 expression. However, as we consistently observe enhanced expression of UCP1 by intermediate concentrations of ATRA in mouse fat cells, we find this difference between mouse and human adipocytes noteworthy.

## Conclusions

In conclusion, we demonstrate that ATRA is a powerful inducer of UCP1 expression in mouse white and brown adipocytes, supporting that ATRA has the capacity to increase the potential for uncoupled respiration in those cells. The increased expression of UCP1 in response to ATRA is mediated by RARs, not PPARδ, and is independent of PGC-1α. We do not find induction of UCP1 gene expression by ATRA in the human adipocytes studied here, but whether this applies to the human UCP1 gene in general remains to be determined. Nevertheless, differences between rodents and humans in terms of regulation of UCP1 expression are highly relevant, as modulation of BAT activity and browning of WAT are being considered as potential anti-obesity targets. More studies comparing rodent and human adipocytes are needed to understand their similarities and differences with respect to regulation of UCP1 expression.

## Methods

### Cell culture

WT and Rb−/− MEFs were propagated and differentiated as previously described [56]. Immortalized PGC-1α+/+ and PGC-1α−/− brown preadipocyte cell lines were obtained from Dr. Bruce M. Spiegelman [43], and C3H10T½ mesenchymal stem cells [36] and 3T3-L1 white preadipocytes [63] were obtained from Dr. Karsten Kristiansen. Brown preadipocyte cell lines and C3H10T½ cells were propagated in Dulbecco’s Modified Eagle’s Medium (DMEM) (Life Technologies) supplemented with 10% foetal bovine serum (FBS) (Life Technologies) and differentiated as WT and Rb−/− MEFs. 3T3-L1 cells were propagated in DMEM supplemented with 10% bovine serum and differentiated as WT and Rb−/− MEFs. Thus, all mouse cells were cultured in the presence of rosiglitazone from day 0 until the time of harvesting. The SGBS white preadipocyte cell line was obtained from Dr. Martin Wabitch [44] and propagated in Advanced DMEM/F12 (Life Technologies) with 10% FBS and 2 mM L-glutamine (Life Technologies). Two days postconfluent cells (designated day 0) were induced to differentiate in Advanced DMEM/F12 with 2% FBS supplemented with 0.86 μM insulin (Roche), 1 μM dexamethasone (Sigma-Aldrich), 0.5 mM 3-isobutyl-1-methylxanthine (IBMX) (Sigma-Aldrich), 1 μM rosiglitazone (Cayman Chemical), 1 μM cortisol (Sigma-Aldrich) and 1 nM 3,3′,5-triiodo-L-thyronine (T_3_) (Sigma-Aldrich). On day 3 the cells were fed the same medium as on day 0. On days 6, 9 and 12 medium contained 2% FBS, 0.86 μM insulin, 1 μM rosiglitazone and 1 nM T_3_. hMADS cells were obtained by Dr. Christian Dani and their propagation and differentiation were carried out as described [45,46] with minor modifications. Briefly, hMADS cells were cultured in low glucose DMEM (Lonza) supplemented with 10% FBS, 2 mM L-glutamine, 10 mM HEPES (Lonza) and 2.5 ng/ml human fibroblast growth factor 2 (Life Technologies). Two days postconfluent cells (designated day 0) were induced to differentiate in low glucose DMEM/Ham’s F12 medium (Lonza) with 10 mM HEPES, 2 mM L-glutamine supplemented with 10 μg/ml transferrin, 0.86 μM insulin, 0.1 μM rosiglitazone, 0.2 nM T_3_, 1 μM dexamethasone and 0.5 mM IBMX. At days 2, 4, 6, 8, 10 and 12 medium was supplemented with 10 mM HEPES, 2 mM L-glutamine, 10 μg/ml transferrin, 0.86 μM insulin, 0.1 μM rosiglitazone and 0.2 nM T_3_. Primary human white subcutaneous preadipocytes (Lonza) were cultured in PBM-2 medium (Lonza). Two days postconfluent preadipocytes (designated day 0) were induced to differentiate with PBM-2 medium supplemented with insulin, dexamethasone, IBMX and indomethacin (all supplied by Lonza) according to the instructions of the manufacturer. On day 3 the cells were refed the same medium as on day 0. On days 6, 9 and 12 cells were refreshed with PBM-2 medium containing insulin and indomethacin. All media described above were supplemented with 50 U/ml penicillin and 50 μg/ml streptomycin, and all cells were cultured at 37°C in humidified atmospheric air with 5% CO_2_, except for hMADS cells that were cultured with 10% CO_2_.

Additional ligands were used in concentrations stated in figures and figure legends and were added from either day −2 and onwards in chronic treatment experiments or from day 8 (mouse cells) or day 12 (human cells) in experiments with acute exposure of mature adipocytes. ATRA, TTNPB and AM580 were purchased from Sigma-Aldrich. Tazarotene, CD1530 and BMS493 were purchased from Tocris Bioscience, and GW501516 was kindly provided by Novo Nordisk A/S. All nuclear receptor ligands were dissolved in dimethyl sulfoxide (DMSO) (Sigma-Aldrich), and dishes not supplemented with ligands were treated with an equal volume of DMSO.

### Reverse transcription-quantitative polymerase chain reaction

Total RNA was purified using TRI Reagent (Sigma-Aldrich). Reverse transcription (RT) and RT-quantitative polymerase chain reaction (RT-qPCR) were performed as previously described [56]. Primers used were: ADRP (mouse), fw-GAATTTCTGGTTGGCACTGT, rev-GACCATTTCTCAGCTCCACTC (80 bp); FABP4 (mouse), fw-TGGAAGCTTGTCTCCAGTGA, rev-AATCCCCATTTACGCTGATG (111 bp); RARβ (mouse), fw-ACAGATCTCCGCAGCATCAG, rev-GCATTGATCCAGGAATTTCCA (76 bp); TBP (mouse), fw-ACCCTTCACCAATGACTCCTATG, rev-ATGATGACTGCAGCAAATCGC (190 bp); UCP1 (mouse), fw-GGCATTCAGAGGCAAATCAGCT, rev-CAATGAACACTGCCACACCTC (151 bp); FABP4 (human), fw-AGCACCATAACCTTAGATGGGG, rev-CGTGGAAGTGACGCCTTTCA (132 bp); RARβ (human), fw-AAGTGCTTTGAAGTGGGAATG, rev-GCTTTTCGGATCTTCTCTGTG (143 bp); TBP (human), fw-CCCGAAACGCCGAATATAA, rev-GAAAATCAGTGCCGTGGTTC (83 bp); UCP1 (human), fw-CCAACTGTGCAATGAAAGTGT, rev-CAAGTCGCAAGAAGGAAGGTA (81 bp).

### Whole cell extracts and immunoblotting

Preparation of whole-cell extracts and immunoblotting were done as described [64]. Antibodies used were against glyceraldehyde 3-phosphate dehydrogenase (GAPDH) (Ab8245, Abcam) and UCP1 (Ab10983, Abcam).

### Statistical analysis

All experiments were repeated at least three times and three dishes were harvested at each time point or treatment for each independent experiment. Data from a representative experiment are presented as mean of the three dishes (+SEM). Statistical significance was determined by Student’s *t*-test. Bonferroni correction was applied when multiple comparisons were carried out.

## Abbreviations

ADRP: Adipose differentiation-related protein; ATRA: All-*trans* RA; BAT: Brown adipose tissue; COUP-TFII: Chicken ovalbumin upstream promoter transcription factor II; DMEM: Dulbecco’s Modified Eagle’s Medium; FABP4: Fatty acid-binding protein 4; FBS: Foetal bovine serum; GAPDH: Glyceraldehyde 3-phosphate dehydrogenase; HPrAD: Primary human white preadipocytes/adipocytes; IBMX: 3-isobutyl-1-methylxanthine; MAPK: Mitogen activated protein kinase; MEF: Mouse embryo fibroblast; PGC-1α: PPARγ coactivator-1α; PPAR: Peroxisome proliferator-activated receptor; RA: Retinoic acid; RAR: RA receptor; Rb: Retinoblastoma gene; RT-qPCR: Reverse transcription-quantitative polymerase chain reaction; T3: 3,3′,5-triiodo-L-thyronine; TBP: TATA-binding protein; TR4: Testicular orphan receptor 4; UCP1: Uncoupling protein 1; WAT: White adipose tissue; WT: Wild-type.

## Competing interest

The authors declare that they have no competing interests.

## Authors’ contributions

MM, MSI, BQ and JBH conceived and designed the experiments. MM, MSI, ALB, SW, CS, JSP, MMN and ASH performed the experiments. All authors analyzed and interpreted the data. MSI prepared the figures. MM, MSI and JBH wrote the paper. All authors read and approved the final manuscript.
